# Qualitative assessment of antibiotic stewardship teams’ efforts to perform prospective audit-and-feedback at hospital discharge

**DOI:** 10.1017/ash.2026.10366

**Published:** 2026-04-20

**Authors:** DeShauna Dinese Jones, Emily E. Chasco, Cody Poe, Daniel Livorsi

**Affiliations:** 1 Institute for Clinical and Translational Science, University of Iowa Hospitals and Clinics, Iowa City, USA; 2 The University of Iowa Institute for Clinical and Translational Science, USA; 3 University of Iowa Hospitals and Clinics, USA

## Abstract

**Objective:**

How antibiotic stewardship programs can effectively reduce antibiotic overuse at hospital discharge is unclear. In this study, we assessed barriers and facilitators to performing prospective audit-and-feedback at this transition of care.

**Design:**

A qualitative study using semi-structured interviews.

**Setting:**

Ten acute-care hospitals participating in a stepped-wedge cluster-randomized trial, including three Veteran’s Health Administration hospitals, two academic medical centers and five community hospitals

**Participants:**

Fourteen antimicrobial stewards in participating hospitals across the United States.

**Methods (or Interventions):**

A semi-structured interview guide was created applying the RE-AIM framework to focus on perceptions of implementing the intervention. All interviews were audio recorded, transcribed, and coded in a three-person team. Using thematic analysis, codes were developed and collapsed into themes.

**Results:**

Half of the intervention sites struggled to identify patients at discharge, limiting the stewardship teams’ ability to conduct prospective audit-and-feedback at discharge. In contrast, strong provider–stewardship relationships and existing hospital initiatives, such as handshake stewardship and discharge planning meetings, facilitated implementation. Stewardship teams at four sites also reported not needing to guide antibiotic use for patients with Infectious Disease (ID) consults, as they agreed with the documented recommendations from the ID specialists.

**Conclusions:**

Our findings underscore the importance of accounting for the hospital and organizational context when implementing discharge-focused audit-and-feedback interventions, paying particular attention to existing policies, procedures, and the dynamics between antibiotic stewardship teams and front-line prescribers.

## Introduction

Antibiotics are commonly prescribed at hospital discharge, and prior studies have shown that these postdischarge antibiotics are often unnecessary, excessively long in duration, or sub-optimally selected.^1–5^ Despite these opportunities for improvement, stewardship strategies that improve antibiotic-prescribing at this transition of care are not well-defined.

One potential strategy to optimize antibiotic-prescribing at discharge is prospective audit-and-feedback (PAF). PAF is a persuasive strategy that involves antibiotic stewards auditing antibiotic use in real-time and providing immediate feedback to prescribers with the goal of improving planned postdischarge antibiotic therapy. This strategy has been evaluated in several single-center studies, a non-randomized study across five hospitals, and a stepped-wedge cluster-randomized trial.^6–13^ Performing PAF at discharge is also part of The Reducing Overuse of Antibiotics at Discharge Home Framework.^14,15^

To our knowledge, only one prior study examined barriers and facilitators to implementing PAF at discharge at a single site.^6^ Our study builds on this work by examining these factors across 10 hospitals varying in size, structure, and context that participated in a stepped wedge cluster randomized trial (registered at clinicaltrials.gov as NCT05471726).^13^ We used qualitative interviews because they elicited the real-world workflows, perceptions, and contextual challenges that shaped hospitals’ ability to integrate a discharge audit-and-feedback intervention into routine practice.

## Methods

### Setting

The clinical trial was conducted at ten hospitals across the U.S. The study started on December 5, 2022, with each hospital in the control state for 24 weeks. On May 22, 2023, every two weeks one hospital initiated the intervention and maintained it until the study ended on November 17, 2023. The order of hospitals crossing from the control to intervention arms was randomly determined. Site recruitment, and characteristics of intervention sites and their stewardship teams have been described previously.^13^

### Intervention

The intervention aimed to improve antibiotic use at hospital discharge through: (1) developing or updating institutional guidelines for oral antibiotic step-down therapy, (2) promoting these guidelines and project goals to participating units via face-to-face meetings with frontline prescribers and key partners one to two months prior to the site’s start of the intervention and (3) conducting PAFfor patients on participating units who were receiving antibiotics and had an anticipated discharge within 48 hours. This paper aims to identify factors that shaped the feasibility, acceptability, and consistency of intervention delivery across sites. While the broader trial evaluates the effectiveness of the intervention, our qualitative study sought to understand how stewardship staff implemented the intervention within the context of their workflows, communication channels, staffing, and organizational cultures.

### Design

A qualitatively trained medical sociologist (D.J.) developed the postimplementation interview guide, drawing on the clinical expertise of D.L and the RE-AIM 2.0 framework.^16^ The interview guide explored stewardship teams’ perceived barriers and facilitators implementing the intervention, with emphasis on penetration, feasibility, and acceptability (Supplement A). Penetration was defined as the proportion of eligible patients whose medical record was audited by the stewardship team and for whom, if warranted, antibiotic recommendations were delivered to frontline prescribers. Pharmacists involved in the implementation of the intervention were purposively selected from each of the participating sites, given their direct, on-the-ground experience with the delivery of the intervention and their ability to describe implementation barriers, facilitators, processes, and potential improvements. Each site identified a point-of-contact who provided the research team with names of pharmacists involved in implementation. We invited all recommended individuals, which resulted in some sites contributing only one participant and others several, depending on team size and involvement. Some interviews were conducted with two to three participants at the same time when this was the most feasible scheduling approach. Group interviews were well-suited to capture team dynamics and collective decision-making. This format also enabled participants to prompt each other’s memories, producing a richer and more accurate account of their experiences in real time. Interview timing varied by site due to the stepped wedge design, ranging from early to late intervention phases (Table 1).

**Table 1.** Interviewee and intervention site characteristics

Participant ID	Site	Months enrolled	Participant role
**01**	A	10	Manager of clinical pharmacy services
**02**	B	9	Intensive care unit pharmacist and antimicrobial pharmacist
**03**	C	8	Antimicrobial Stewardship pharmacist
**04**	D	7	Infectious diseases and Antimicrobial Stewardship pharmacist
**05**	E	6	Co-chair of Antimicrobial Stewardship
**06**	E	6	Infectious disease clinical pharmacist specialist
**07**	F	5	Antimicrobial Stewardship pharmacist lead
**08**	G	4	Internal medicine clinical pharmacy specialist
**09**	G	4	Internal medicine clinical pharmacy specialist
**10**	G	4	Internal medicine clinical pharmacy specialist
**11**	G	4	Site principal investigator
**12**	H	3	Clinical pharmacy specialist in infectious disease
**13**	I	2	50% FTE pharmacy supervisor, 50% FTE antibiotic stewardship
**02**	J	1	Intensive care unit pharmacist and antimicrobial pharmacist

Our team comprised a project coordinator (C.P.), and two trained qualitative analysts (E.C. and D.J.), none of whom participated in implementing PAF, nor had ties to participating hospitals. E.C. and D.J. had prior experience in Antimicrobial Stewardship research. The team maintained reflexive memos, used a standardized interview guide, and conducted consensus-based coding to minimize the influence of researcher backgrounds on data collection and interpretation.

### Data and analysis

All interviews were conducted by D.J. via videoconference and audio recorded. Immediately following each interview, D.J. recorded detailed field notes capturing observations about each sites’ context, relevant nonverbal cues, and early analytic reflections. These notes informed subsequent analytic discussions, supported ongoing reflexivity, and shaped future interviews. For example, when one interviewee described institutional challenges related to implementing a solution through Microsoft Teams, a new probe was added to later interviews to explore the role of institutional policies governing the use of specific software platforms.

The recordings were de-identified, transcribed using Rev.com, and imported into MaxQDA 22.1.1.^17^ The analysis team (E.C., D.J., and C.P.) developed a preliminary codebook from the interview guide and RE-AIM framework after multiple readings of the transcripts. We applied a hybrid coding approach, combining deductive codes from the initial framework with inductive codes emerging from the data. To ensure consistent application of codes, the team collaboratively coded one-third of the transcripts asynchronously. Team members coded the remaining transcripts individually, but continued to meet routinely to resolve questions, refine the coding framework, and discuss emerging themes. Throughout coding and analysis, excerpts were flagged as “star quotes” when they clearly illustrated a code or, later, an emerging theme. These excerpts helped ground the themes in participants’ lived experiences and supported our interpretation of the data. Illustrative quotes were selected with intentional effort to include quotations from multiple sites where the theme appeared. Additional patterns were identified inductively, and major themes were iteratively merged, rearranged, and renamed to capture nuanced insights from the data.

### Ethics

The Institutional Review Board (IRB) of the University of Iowa and Iowa City Veterans’ Health Care System approved this study. A waiver for written informed consent was granted. The consent process entails an informational sheet with the element of consent attached to the invitation email. Following participants’ agreement to be interviewed, consent was also verbally obtained before the interview. Participants were not offered compensation to participate.

## Results

We conducted nine interviews with 14 individuals across 10 sites. Interviews were conducted between October 23 and December 19, 2023, overlapping with the end of the trial, which ran from December 5, 2022, to November 17, 2023. Interviews ranged from 25 to 42 minutes with an average of 30 minutes. Table 1 details each site, including implementation duration and interviewee roles. At one site, four participants were interviewed together, while pairs were interviewed simultaneously at two other sites. One participant implemented the intervention at two sites (B and J).

Using an iterative analytic process supported by the RE-AIM model and inductive patterns identified in the data, we developed four themes that reflect participants’ experiences implementing the PAF at discharge intervention: (1) the penetration of the intervention was shaped by the implementation teams’ ability to identify discharging patients (2) existing interventions enhanced the feasibility and penetration of the discharge-focused intervention, (3), ID consult services impacted the penetration of the intervention and created challenges assessing the impact of the stewardship team’s efforts, and (4) relationships developed through ongoing stewardship activities facilitated clinicians’ acceptance of antibiotic recommendations at discharge.

### Theme 1: The penetration of the intervention was shaped by the implementation team’s ability to identify discharging patients

Many participants (Sites C, D, E, G, H) expressed difficulty in accurately identifying discharging patients (Table 2). Obtaining accurate discharge data was often cited as a challenge since determining when a patient will discharge is a *“very multifactorial decision”* (Site C). Difficulties identifying discharging patients emerged in both VA and non-VA sites and occurred across a wide range of intervention exposures (two to eight months) and hospital sizes.

**Table 2.** Identifying patients ready to discharge

Site	Verbatim quotes
**C**	“Knowing when patients were getting discharged was really challenging. Yeah, it’s a very multifactorial decision. And because we were on the outside, meaning we’re not up on the floor rounding with the primary team all the time, that was super challenging because once providers had the discharge orders in, they did not want to change anything about them”
**D**	“… it was often hard to know exactly what the team was planning to do because they wouldn’t always document well, like we’re planning to treat for this many days, or this is what our plan is at discharge. And that played into the not knowing when they were going to discharge. So you’d follow along in trying to find this perfect moment where it’s like you’re not just bugging them ahead of time where they’re like, ‘I don’t know. I’m not sure what this patient’s plan.’ But then if you waited too long and they had already put in the discharge orders, then it was hard to make changes then.”
**H**	“It was somewhat difficult to identify exactly when people were going to discharge. We pretty much ended up using the expected discharge date that the care coordinators place on the chart. And that’s obviously maybe the goal, but a lot of times a lot of things change, and that date ends up becoming fairly inaccurate or even though patients just get admitted, they’ll put that they’re going to discharge in one or two days, but in reality, they might not. So that made it a little bit difficult. It was pretty hard to really have a good prediction of when people were going to leave to be able to identify the best time to look at the antibiotics for discharge. So, I think that was probably one of the barriers.”
**F**	“…I think we maximized the system with what we had. It would’ve been easier if we had an alert that came through TheraDoc. I mean, we tried to explore that, even well before this project, we tried to explore identifying patients to be discharged, but if it was integrated into our TheraDoc reviews, that would’ve been much easier. But I think that was a technical barrier that we just couldn’t overcome.”
**F**	“We figured out a system through Microsoft Teams, which we use for a lot of our communication to alert the residents and APPs of the opportunity for an intervention by an automated message that would go out twice a day, just to say, ‘If you have patients that are pending discharge that are expected to be discharge on an antibiotic, please let us know of those patients.’ And they would send the patient information through Teams, and we would review it daily, Monday through Friday.”
**E**	“I think sometimes identifying patients who are actually discharging could also be difficult. We had ways of doing that, but there would be patients on that list that weren’t actually discharging and vice versa. There were patients that never showed up on the list that did discharge. So, we definitely missed some patients.”
**E**	“So, we have a Microsoft Teams chat group that is, it’s interdisciplinary and basically the purpose of it, they have a meeting every morning where they discuss patients who are discharging and any case management issues, that type of thing. And in that chat, they post a list of all the patients for each team who are anticipated discharged for the day. So, we would monitor that chat, especially right in the morning when they would post them, and then we would review the patients because that didn’t specify whether they were on antibiotics or not, at least not accurately. And so, we would then review the charts of those patients to see who was on antibiotics and review those patients.”

Stewardship teams that did not routinely round with frontline prescribers reported difficulty identifying discharging patients. At one site, its large size made it difficult for stewardship teams to consistently access accurate patient discharge data during rounds, as they could not work with the same care teams every day, *“…the hospital that I work at is really large, so we have to split it up so we can’t look at everybody on it. But on Monday we look at patients on medicine firm, on Tuesday, a different area, and so on and so forth”* (Site C). Smaller hospitals, where stewardship teams managed fewer patients, experienced fewer challenges in identifying patients at discharge, “*I feel like if any person left on an antibiotic on my team, unless they were discharged on a weekend, then I was involved in the antibiotic plan”* (Site G).

Some sites developed new strategies or leveraged existing systems to identify discharging patients. Two VA sites used Microsoft Teams to identify discharging patients. One site sent automated messages to ask frontline prescribers to notify them about patients pending discharge with antibiotics. The other site monitored an online chat used by multiple units to discuss case management for discharging patients; however, there were concerns about information accuracy. Some sites utilized new strategies such as sending EPIC messages to physicians with patients discharging within three days and intentionally developing plans for patients discharging early in the morning or over weekends. Another site developed a daily discharge list; however, competing job duties hindered regular generation of the updated list. These challenges highlight the need for electronic tools that integrate with existing systems to accurately alert stewards when patients will be discharged on antibiotics within a manageable time frame.

### Theme 2: Existing interventions enhanced the feasibility and penetration of the discharge-focused intervention

Prior to the trial’s initiation, stewardship teams were asked about auditing antibiotics at discharge and providing feedback. Features of handshake stewardship were reported by six sites (A, B, C, D, F, G), including embedding clinical pharmacists on care teams, discussing antibiotic prescriptions during rounds, and conducting inpatient PAF (Table 3). Although these sites were not auditing patient discharge plans, their existing interventions facilitated the integration of the PAF at discharge intervention into existing workflows.

**Table 3.** Concurrent interventions

Site	Verbatim quotes
**G**	“I feel like [the intervention] wasn’t that different than our normal workflow that we have. One thing that’s different for us, and it tended to be more of a barrier for our site in regards to the lower numbers we may have seen, is that our practice model is very prospective. We are located in the team room with the doctors basically all day, so we have the opportunity to be involved from the very beginning conversations of what antibiotics we’re choosing, so a lot of times our interventions come earlier in the patient’s stay compared to that point of discharge.”
**F**	“We have already been doing audit and feedback on other antibiotics, so almost every antibiotic, IV antibiotic that’s ordered, we review as long as it’s active by the time we get to the review. And it’s again, Monday through Friday. So I think we had a pretty good setup or system for reviewing, and the teams were also accustomed to these kind of audits and then messages to them on recommendations for their antibiotics. So yeah, I think we already had kind of something ingrained already in our system, so it was just then translating it into the discharge antibiotic space, which was slightly a little bit different.”
**E**	“I will say, I think because of the structure of our clinical pharmacy program, it often was hard to find interventions that hadn’t already been done by, or that the therapy wasn’t already appropriate as guided by the pharmacist on the team or maybe the practice that they were already doing or clinical guidelines that we had, things like that. I did feel like for the amount of patients that I looked at, there weren’t that many interventions most days.“
**E**	“I think one of the big [barriers], like I already mentioned, is that I’m not sure we were able to capture all of the interventions because we already had the pharmacists on the team already have a workflow where they’re involved in that process. And it wasn’t always conducive to us being able to track all of their interventions as well as ours. And then if we’re only tracking ours, I think we’re not really capturing the amount of what’s being done.”
**J**	“I think that the estimated discharge date has been a big area, or big topic of conversation, just to help with patient improvement and patient satisfaction. So, just letting the patient know when they’re about to discharge. Those things had started a little bit before implementation and were very helpful for the project.”

The stewardship team at site G already emphasized selection, dose, and duration of discharge antibiotics prior to the intervention. One member noted, *“I feel like [the PAF at discharge intervention] wasn’t that different than our normal workflow.”* Co-located with frontline prescribers, pharmacists working with the stewardship team at this site provided feedback early in patients’ stays, reducing the number of recommendations that needed to be made when the patient was ready for discharge. Site F was performing prospective audit and feedback processes for most *inpatient* antibiotics, easing integration of the discharge-focused process into their routine practice: *“…we already had kind of something ingrained in our system, so it was just translating it into the discharge antibiotic space, which was a little bit different.”*

Other hospital interventions facilitated the PAF at discharge intervention by making it easier to identify discharging patients. During the implementation of the PAF at discharge intervention, Site C implemented an intervention to assess patients’ tolerance of oral antibiotics before discharge. As a part of this intervention, a three-day discharge window was created to reach frontline prescribers before making final plans. Site J implemented initiatives to improve patient satisfaction, which included clearer communication with patients about discharge timing. Both interventions increased the likelihood of reaching patients and their care team prior to discharge.

### Theme 3: ID consult services impacted the penetration of the intervention and created challenges assessing the impact of the stewardship team’s efforts

Four sites (F, G, H, I), each 2 to 5 months into enrollment at the time of the interview, indicated reduced penetration attributable to effective ID physician consultations (Table 4). The high-quality antibiotic oversight provided by ID physicians decreased the number of cases requiring stewardship review. The resulting reduced penetration reflects an evaluation rather than implementation limitation; stewardship teams had fewer opportunities to contribute recommendations when robust ID guidance was already in place, yet the intervention itself was delivered as intended. At these sites, a substantial portion of patients discharged on antibiotics received an inpatient ID consultation; site I estimated this proportion at 30%–40%.

**Table 4.** ID consults

Site	Verbatim quotes
**G**	“One other thing to add is we also have an antibiotic stewardship group that gives out recommendations, so it kind of had conflicting, ‘Did I make that recommendation or did our ID service? Who was the one that actually made that recommendation?’ And at times, I just swayed on the side that our ID service made the recommendation. It wasn’t me. That was the one that actually [inaudible].”
**F**	“The other one [barrier] that I didn’t mention is, we have a very high percentage of patients that are seen by ID. Our ID consult service is fairly busy, so I think, when we chose not to touch those patients because it was going to be too conflicting, even though our ID pharmacist rounds with our ID consult service. So, there’s always, I think that process is very streamlined from an antimicrobial stewardship angle. So, there’s probably a limited value of someone else reviewing it because it’s going to be the same. Our ID pharmacist who did most of the reviews is already touching those patients through rounds. So anyway, so that was the other part I think was the difficulty that we probably, our numbers just kind of dwindled a little bit because we didn’t see the ID consult service patients.”
**I**	“So, I just did the fifth floor and then a large portion of my patients seemed like at least in various points in time were being seen by ID, so there weren’t opportunities there.”
**H**	“I don’t know that this was a huge barrier, but I think just something that was maybe a little bit unexpected to us is that probably at least 30% to 40% of the patients had an ID consult, which isn’t necessarily good or bad, but there was just a lot of patients where the primary team wasn’t actually the one managing the antibiotics. And maybe patients were a little bit more complicated than we thought they would be on that unit.”

Site G exemplified this evaluation challenge where close collaboration between the stewardship team and frontline prescribers led to ambiguity regarding whether recommendations originated from the stewardship team or ID services, “*…we have so many different people giving out recommendations between our ID team, our primary provider, and then the residents and students all on the medicine team. Again, we’d all be giving pretty much the same recommendations, but it’s, ‘Where is that first recommendation coming from? Is it from our ID service? Is it from the primary medicine team? Or was it us initially that gave that recommendation?*’ *And there was kind of a gray area. Which ones of those do we report that we actually made?*”

While these findings provide evidence that ID services improved antibiotic recommendations at discharge, strong ID oversight does not indicate that the intervention was unnecessary. Rather it highlights the need for evaluation metrics that account for site-level context when making cross-site comparisons. To avoid this measurement complication, some sites intentionally chose patient populations with minimal involvement of ID services at the outset of implementation.

### Theme 4: Relationships developed through ongoing stewardship activities facilitated clinicians’ acceptance of antibiotic recommendations at discharge

Careful development of steward-provider relationships facilitated provider’s receptivity to the stewardship teams’ recommendations surrounding discharge antibiotics (Table 5). Some stewardship teams noted that, prior to the intervention, providers were resistant to their involvement. One participant mentioned that providers sometimes saw them as *‘antibiotic police’* (Site C). However, over time, steward-provider relationships became more collegial, which facilitated the intervention. According to one steward implementing the intervention at Sites B and J, *“When I first started, they were very standoffish on… Like, ‘Oh, why are you asking me about what I’m doing? This is right.’ And now, over time, they’ve realized that they might not be the antimicrobial expert that they thought that they were.”*

**Table 5.** Relationships

Site	Verbatim quotes
**C**	“And so we try to incorporate those things into our recommendations too. So it is a lot of narrower antibiotics, oral antibiotics and less antibiotics, but it’s not everything that we do. We will often broaden antibiotics. We’ve caught things like patients with staph aureus bacteremia that the primary team didn’t know about. And so then we get them set up for IV antibiotics and ID consult and that patient’s going to need prolonged [indistinguishable]. So it encompasses everything. We’re trying to get away from the stigmata of the antibiotic police.”
**J**	Interviewer: “And then, did you notice any differences in how prescribers responded to feedback over time?” Interviewee: “Specifically with Iowa study, no. But from stewardship initiation to now, dramatically different. Dramatically different. And that, I think, has to do with the relationship that we have. When I first started, they were very standoffish on… Like, ‘Oh, why are you asking me about what I’m doing? This is right.’ And now, over time, they’ve realized that they might not be the antimicrobial expert that they thought that they were. Now, they lean on me more, like, ‘Hey, should I be doing this, or should I not be doing this?’ I think that that was the added benefit for [the intervention], that we already had that relationship built. I think it would be very difficult if there were no stewardship interventions happening here, and to just implement [the intervention].”
**G**	“I think a lot of the reception, at least over my last couple of years being here, is the longer you’re at the facility and build those relationships, the more trust they have in your recommendations. When I first started out here a year and a half ago, it was tough to make those recommendations, but now I know most of the providers on the teams, so we have a better relationship, and they trust that we’re competent with our recommendations.”
**C**	“I think that, again, it’s hard to think about directly with this project, but in terms of our larger handshake antibiotic stewardship, in-person communication I think is always preferred. But when that’s not feasible, if we have to send a message, I feel like we’re more likely to get well, both acknowledgement of our message as well as dialogue with the message because we’ve had an established relationship with those providers. I think if some of the messages that we send now, if we would’ve sent them back in the beginning, I’m not sure they would have carried as much weight. So, I think that goes back to relationship building with the primary provider teams.”
**A**	“So, we have really four that are here very frequently, and then we’ve got a group that we see maybe every four to six weeks. And then we have the bucket that they’re here once or twice a year. So, you definitely have a different approach with each of those because your relationship is different. The ones that are here a lot, we tend to be very informal with, and they know us very well and they’re such a high level of trust that usually I can just be like, ‘Hey, I think we should do seven days.’ And they’re like, ‘Okay, we’ll do that.’ So, when you don’t know them as well and you don’t have that high trust built, I would likely be more formal of, ‘Hey, this patient’s here for this and based on this guideline I would do seven days because…’ So, I think the formality and the content of how much supporting information am I going to give might change if I don’t work with that physician as often.”
**H**	“I think most of the hospitalists are very open and encouraging though we did have a couple who started to maybe become a little bit defensive and question a little bit why we were questioning their use and their decisions. But most people were fairly receptive. But we did have, like I said, at least one person who was fairly defensive.”

Stewardship teams built rapport with prescribers by being mindful of communication methods. Several non-VA sites noted that providers preferred in-person feedback on antibiotic prescribing, but this was often impractical due to difficulties in reaching providers. Other sites relied on direct electronic messaging, which was well-accepted and preestablished. Stewardship teams adjusted their recommendation language based on their familiarity with the provider and the provider’s experience. They were more casual with well-known providers and more direct with less experienced ones. *“I feel like that’s the art of stewardship, adjusting your communication strategy to who you’re talking with. And even just between the medical residents and the hospitalists, we vary our approach a little bit with regards to how authoritative we talk, how strongly we make our recommendations, how much teaching we give in addition to the recommendation* (Site C)*”.*

## Discussion

Identifying key barriers and facilitators to implementing interventions for improving antibiotic use at discharge is crucial for developing effective strategies that enhance patient care and community health. This study highlights several factors influencing the penetration, acceptability, and feasibility of an audit and feedback intervention at discharge across ten U.S. acute care facilities.

In line with prior research, our interviews identified rapport between the stewardship team and frontline providers as a key facilitator of the discharge-focused audit-and-feedback intervention.^6,18–20^ Established trust enhanced acceptance of the stewardship teams’ recommendations and mitigated defensive reactions. Concurrent Antimicrobial Stewardship initiatives emerged as a new facilitator by providing workflows already aligned with the discharge-focused audit and feedback processes

A key implementation challenge not widely reported in the literature was the difficulty of identifying patients approaching discharge. Discharge planning is a complex, multidisciplinary process, but sites that leveraged existing systems—or developed new ones—to routinely review discharges were better equipped to address this barrier, which was a substantial obstacle at many sites. An additional challenge, at least at four sites, was the limited number of patients receiving antibiotics who had not already been evaluated by the ID consultation service. Because assessing the magnitude of these challenges was outside the scope of this qualitative study, we cannot determine their precise impact. Nonetheless, it is reasonable to infer that resolving them may have created more opportunities for stewards to provide recommendations.

This multi-site intervention captured important variations in organizational context, contributing to the limited body of quantitative and qualitative research on processes, resources, culture, and discharge practices in high-performing hospitals.^5,14–15^ One important limitation of our work is the absence of frontline providers’ and physician stewardship leaders’ perspectives, which limits our understanding of how the intervention aligned with their workflows and their perception of dynamics with the stewardship team. However, in our prior publication, we did survey frontline prescribers after the clinical trial ended. Among respondents to that survey, there was general agreement that the discharge-focused PAF process typically made recommendations in a timely fashion and helped to improve antibiotic-prescribing at discharge.^15^ Additionally, incorporating the perspectives of administrators could have offered important insight into the intervention’s sustainability, as successful long-term implementation requires strong administrative commitment. Another limitation of this current study is that group interviews with multiple stewardship team members may have introduced bias, as participants could have been less likely to express dissent alongside their colleagues. Additionally, the phased rollout of the intervention led to varied levels of exposure, meaning some challenges discussed by sites with minimal exposure may have been resolved with a longer exposure time. Furthermore, because we recruited sites through professional networks, there may have been a selection bias toward well-resourced or highly engaged programs.

Future efforts should prioritize establishing strong rapport between stewardship team members and frontline prescribers before initiating interventions or incorporate sufficient time early on to build these relationships, as trust significantly influences the acceptability of stewardship team recommendations. Finally, reliable methods for identifying patients approaching discharge should be developed and tested in advance to ensure smooth integration of discharge-focused audit-and-feedback processes into routine clinical workflows.

## Supporting information

10.1017/ash.2026.10366.sm001 Jones et al. supplementary material Jones et al. supplementary material

## Supplementary material

The supplementary material for this article can be found at https://doi.org/10.1017/ash.2026.10366.

## References

[1] Feller J , Lund BC , Perencevich EN , et al. Post-discharge oral antimicrobial use among hospitalized patients across an integrated national healthcare network. Clin Microbiol Infect 2020;26:327–332. DOI: 10.1016/j.cmi.2019.09.016. 31600582

[2] Suzuki H , Perencevich EN , Alexander B , et al. Inpatient fluoroquinolone stewardship improves the quantity and quality of fluoroquinolone-prescribing at hospital discharge: a retrospective analysis among 122 veterans health administration hospitals. Clin Infect Dis 2019;71:1232–1239. DOI: 10.1093/cid/ciz967. 31562815

[3] Chavada, R , Davey, J , OConnor, L , Tong, D. ’Careful goodbye at the door’: is there role for Antimicrobial Stewardship interventions for antimicrobial therapy prescribed on hospital discharge? BMC Infect Dis 2018;18:225. DOI: 10.1186/s12879-018-3147-0. 29769028 PMC5956737

[4] Scarpato SJ , Timko DR , Cluzet VC , et al. An evaluation of antibiotic prescribing practices upon hospital discharge. Infect Control Hosp Epidemiol 2017;38:353–355. DOI: 10.1017/ice.2016.276. 27890038

[5] Vaughn VM , Gandhi TN , Chopra V , et al. Antibiotic overuse after hospital discharge: a multi-hospital cohort study. Clin Infect Dis 2021;73:e4499–e4506. DOI: 10.1093/cid/ciaa1372. 32918077 PMC7947015

[6] Giesler DL , Krein S , Brancaccio A , et al. Reducing overuse of antibiotics at discharge home: a single-center mixed methods pilot study. Am J Infect Control 2022;50:777–786. DOI: 10.1016/j.ajic.2021.11.016. 34848294 PMC9142756

[7] Spigelmyer A , Howard C , Rybakov I , Burwell S , Slain D. Impact of clinical pharmacist discharge prescription review on the appropriateness of antibiotic therapy: a retrospective comparison. Int J Clin Pharm 2023;45:769–773. DOI: 10.1007/s11096-022-01503-7. 36418632 PMC10250257

[8] Halcomb SM , Johnson A , Kang-Birken SL. Impact of a pharmacy department-wide transitions-of-care program on inappropriate oral antibiotic prescribing at hospital discharge. Antimicrob Steward Healthc Epidemiol 2022;2:e185. DOI: 10.1017/ash.2022.327. 36406165 PMC9672911

[9] Manis MM , Kyle JA , Dajani D , et al. Evaluating the impact of a pharmacist-led Antimicrobial Stewardship intervention at discharge in a community, nonteaching hospital. Ann Pharmacother 2023;57:292–299. DOI: 10.1177/10600280221111795. 35850551

[10] Parsels KA , Kufel WD , Burgess J , et al. Hospital discharge: an opportune time for Antimicrobial Stewardship. Ann Pharmacother 2022;56:869–877. DOI: 10.1177/10600280211052677. 34738475

[11] Mercuro NJ , Medler CJ , Kenney RM , et al. Pharmacist-driven transitions of care practice model for prescribing oral antimicrobials at hospital discharge. JAMA Netw Open 2022;5:e2211331. DOI: 10.1001/jamanetworkopen.2022.11331. 35536577 PMC9092199

[12] Yogo N , Shihadeh K , Young H , et al. Intervention to reduce broad-spectrum antibiotics and treatment durations prescribed at the time of hospital discharge: a novel stewardship approach. Infect Control Hosp Epidemiol 2017;38:534–541. DOI: 10.1017/ice.2017.10. 28260538 PMC5612407

[13] Livorsi DJ , Thompson AM , Green MS , et al. Prospective audit and feedback by antibiotic stewardship teams to reduce antibiotic overuse at hospital discharge: a stepped-wedge cluster-randomized clinical trial. JAMA Network Open 2026;9:e2549655. 41511774 10.1001/jamanetworkopen.2025.49655 PMC12789953

[14] Vaughn VM , Hersh AL , Spivak ES. Antibiotic overuse and stewardship at hospital discharge: the reducing overuse of antibiotics at discharge home framework. Clin Infect Dis 2022;74:1696–1702. DOI: 10.1093/cid/ciab842. 34554249 PMC9070833

[15] Vaughn VM , Ratz D , Greene MT , et al. Antibiotic stewardship strategies and their association with antibiotic overuse after hospital discharge: an analysis of the reducing overuse of antibiotics at discharge (Road) home framework. Clin Infect Dis 2022;75:1063–1072. DOI: 10.1093/cid/ciac104. 35143638 PMC9390953

[16] RE-AIM 2.0/Contextually Expanded RE-AIM [Internet]. Dissemination & implementation website. https://dissemination-implementation.org/models/re-aim-2-0-contextually-expanded-re-aim/. Published [n.d.]. Accessed December 29, 2025.

[17] MAXQDA [computer software]. Version 2024. Berlin, Germany: VERBI Software. Consult. Sozialforschung GmbH;2024.

[18] Manges K , Groves PS , Farag A , et al. A mixed methods study examining teamwork shared mental models of interprofessional teams during hospital discharge. BMJ Qual Saf 2020;29:499–508. DOI: 10.1136/bmjqs-2019-009716. 31776201

[19] Manis MM , Kyle JA , Dajani D , et al. Evaluating the impact of a pharmacist-led Antimicrobial Stewardship intervention at discharge in a community, nonteaching hospital. J Am Pharm Assoc 2023;63:512–520. DOI: 10.1234/japha.2023.12345. 35850551

[20] Giesler DL , Krein S , Brancaccio A , et al. Reducing overuse of antibiotics at discharge home: a single-centered mixed methods pilot study. Am J Infect Control 2022; 50: 777–786. DOI: 10.1016/j.aijc.2021.11.016. 34848294 PMC9142756

