# Supplementary material for: Qualitative assessment of antibiotic stewardship teams’ efforts to perform prospective audit-and-feedback at hospital discharge
Source: Antimicrob Steward Healthc Epidemiol. 2026 Apr 20;6(1):e113. doi: 10.1017/ash.2026.10366 (PMC13104581; doi:10.1017/ash.2026.10366)
Supplement: Jones et al. supplementary material [file S2732494X26103660sup001.docx]

# **Supplement A. Post-Intervention Semi-Structured Interview Guide**

1. What is your primary role at your facility?
2. How long have you worked at your current facility?
3. What people or roles did you work with to implement this audit-and-feedback intervention?
4. Tell me your general perceptions of the audit-and-feedback process your team implemented at hospital discharge.
5. Was the process feasible to implement? Why or why not?
   1. Did your team need to make any modifications to the guidelines surrounding oral antibiotic step-down therapy?
   2. How did your team decide what inpatient services to include in the intervention?
   3. What activities (e.g., presentations, posters, screen savers) did the stewardship team do prior to the start of the intervention to promote the new audit-and-feedback process?
6. What (other) barriers did you encounter when implementing the audit-and-feedback process at hospital discharge?
   1. How did you address these barriers?
   2. Did you need to modify your audit-and-feedback process to better address these challenges?
7. Were there certain individuals or existing hospital processes that facilitated your implementation of this new discharge-focused process?
8. Do you think the process is sustainable?
   1. What do you think needs to be done at your facility to improve the sustainability of the audit-and-feedback process?
9. The next questions are going to focus on the feedback your team gave to frontline prescribers. How did your team usually approach frontline prescribers to provide feedback on patients being discharged on antibiotics?
   1. How often was the feedback given in-person? Via phone? Any other method?
   2. How often were you able to provide feedback before the patient was discharged?
   3. What could have been done differently to increase the number of patients for whom you were able to provide feedback?
   4. Over time, did you modify how you provided feedback?
   5. Did your approach vary from prescriber to prescriber?
10. Overall, do you think patient care was improved by this new audit-and-feedback process?
    1. What did frontline prescribers think of this audit-and-feedback process?
    2. Did you notice differences in how prescribers responded to feedback?
    3. How did prescribers’ perceptions of the process change over time?
11. If the project were to start over, what would you change or do differently next time?
12. After participating in this program, how will your approach to stewardship change?
    1. What major opportunities for improved antibiotic-prescribing at hospital discharge do you see?
    2. How do you plan to address these opportunities?
13. Do you plan to continue this same intervention in some form?
14. Is there anything else you think our study team should know about anything we’ve discussed today?
